# Oxidant-Antioxidant Balance in the Blood of Patients with Chronic Obstructive Pulmonary Disease After Smoking Cessation

**DOI:** 10.1155/2013/897075

**Published:** 2013-09-05

**Authors:** Alina Woźniak, Dariusz Górecki, Michał Szpinda, Celestyna Mila-Kierzenkowska, Bartosz Woźniak

**Affiliations:** ^1^Collegium Medicum of Nicolaus Copernicus University, Karłowicza 24, 85-092 Bydgoszcz, Poland; ^2^Specialist Family Medicine Center, Przesmyk 2/4, 87-100 Toruń, Poland; ^3^Department of Normal Anatomy, Collegium Medicum of Nicolaus Copernicus University, Karłowicza 24, 85-092 Bydgoszcz, Poland; ^4^Department of Neurosurgery, Stanisław Staszic Specialist Hospital, Rydygiera 1, 64-920 Piła, Poland

## Abstract

The effect of smoking cessation on the oxidative stress in patients with chronic obstructive pulmonary disease (COPD) was assessed. We recruited 73 smokers with COPD (study group), whose blood was analysed before smoking cessation, after the 1st, 2nd, and 3rd months of abstinence, 35 healthy nonsmokers (Control I), and 35 smokers with COPD (Control II). Blood was taken once in Control I and 4 times (every month) in Control II. In the study group conjugated dienes (CDs) level in plasma and erythrocytes before smoking cessation was 3 and 6.5 times higher than in Control I, respectively (*P* < 0.001), while thiobarbituric acid-reactive substances (TBARS) level was 89% (*P* < 0.001) and 51% higher (*P* < 0.01), respectively. Superoxide dismutase (SOD) activity was 40% higher (*P* < 0.05) while glutathione peroxidase (GPx) was 41% lower (*P* < 0.001) than in Control I. In Control II, the similar differences as compared to Control I were observed throughout the study. Smoking cessation resulted in decrease of CDs, TBARS, and SOD and GPx increase, with no changes in catalase and vitamins A and E. COPD is accompanied by oxidative stress. A three-month tobacco abstinence facilitated restoring the oxidant-antioxidant balance systemically, but it did not affect spirometric parameters.

## 1. Introduction

Chronic obstructive pulmonary disease (COPD) is the most frequent chronic respiratory disease. It is currently the 4th most common cause of death in the USA and Europe, after cardiovascular diseases, cancer, and road traffic injuries. In light of the increasing number of smokers in the developing countries, particularly in China, COPD is prognosticated to become the 3rd most common cause of death globally by the year 2020 [1]. The main characteristic of COPD is a progressive, irreversible narrowing (obturation) of the bronchi that obstructs the air passage through the bronchi to the lungs. As a result, the lungs lose their elasticity. The disease has an inflammatory background, which is caused by the inhalation of harmful dust and gases [2]. Apart from inflammatory reactions, the domination of proteinases over antiproteinases [3] and oxidative stress [4] are also important factors in the pathogenesis of COPD. It has been proven that the incidence of COPD is strictly correlated with the addiction to smoking tobacco [1, 5, 6]. Toxic derivatives of the metabolism of oxygen present in tobacco smoke, the so-called reactive oxygen species (ROS), may be the cause of oxidative stress, which may be the background of the development of COPD, as hypothesized by some researchers [7]. Oxidative stress in cells and tissues is induced by the imbalance between the generation and removal of ROS. ROS derived from inflammation-inducing cells (neutrophils, macrophages), large numbers of which migrate to the lungs, also play an important role in the oxidant-antioxidant imbalance observed in the course of COPD [7]. The increased ROS generation in the fluid layer on the surface of pulmonary alveolar endothelium may also be a consequence of the increasing content of free iron in the respiratory tract of cigarette smokers [8]. Disturbances in the oxidation-reduction processes in the course of COPD occur not only in the lungs. Increased ROS generation is also observed in the peripheral blood. In numerous articles, the authors indicate an increased (O_2_^•−^) generation by neutrophils in the peripheral blood of patients with COPD exacerbation [9]. Another source of (O_2_^•−^) and H_2_O_2_ in COPD patients is xanthine oxidase. The increased activity of this enzyme is probably caused by an increased expression of proinflammatory cytokines [10], such ROS react with nucleic acids, lipids, and proteins, leading to the damage of lung cells and extracellular structures. They may also induce remodelling of the extracellular matrix of the lungs, as well as cell apoptosis and proliferation, or may interfere with cellular respiration and repair and immune mechanisms, as well as hinder the maintaining of surfactant and antiprotease protection [11].

 One of the results of increased ROS generation is increased lipid peroxidation. It involves free radical chain reactions leading to the decomposition of polyunsaturated fatty acids, constituting, for example, the components of cell membranes [12]. In this process, once a hydrogen atom is detached from a polyunsaturated fatty acid molecule, a reconfiguration of double bonds occurs and leads to the generation of conjugated dienes (CDs) [13, 14]. Among the secondary products of lipid peroxidation, generated as a result of further reactions, (e.g., *β*-elimination and decomposition of polyunsaturated fatty acid derivatives), are aldehydes, mainly malondialdehyde (MDA) [15]. Aerobic organisms have evolved defence mechanisms that protect them against the results of the generation of ROS and their reactions with cell components. The mechanisms are organized in a system of antioxidant enzymes and nonenzymatic ROS scavengers. The main antioxidants are superoxide dismutase (SOD), catalase (CAT), and the glutathione- (GSH-) related system, including glutathione peroxidase (GPx) [16]. SOD catalyses the dismutation of the superoxide anion (O_2_^•−^) to oxygen and hydrogen peroxide (H_2_O_2_). The removal of H_2_O_2_ is performed by CAT and GPx [17]. Among the nonenzymatic low molecular weight antioxidants are, for example, vitamins A, E, and C [18].

 The most recent studies confirm that the incidence of COPD is related to oxidative stress, which, in the case of this disease, may be caused not only by cigarette smoke, but also by hypoxia, infections, inflammations, and ageing [4]. New strategies of COPD treatment will therefore be based on, for example, mitigating oxidative stress. Smoking cessation remains the most effective therapeutic intervention in patients with COPD [19]. In the medical literature, there is scarce information on the effect of tobacco abstinence in patients with COPD on the markers of oxidative stress, and the available results are ambiguous. Therefore, we decided to analyse the effect of giving up smoking on the activity of SOD, CAT, and GPx, the levels of CDs, and thiobarbituric acid-reactive substances (TBARSs), as well as the quantity of vitamins A and E in the peripheral blood of patients with COPD. Another aim of the study was to compare the levels of the determined markers of oxidative stress in the peripheral blood of COPD patients who had given up smoking with the levels of those markers in healthy nonsmokers and in COPD patients who had not given up smoking. Finally, we assessed the usefulness of the determined markers of the oxidant-antioxidant balance in the diagnostics of COPD.

## 2. Materials and Methods

 The study was attended by 73 patients with COPD, aged from 25 to 72 (mean age = 48.9 ± 11.7 years), treated at the Specialist Family Medicine Center in Toruń, Poland (Table 1). The patients had been smoking at least 10 cigarettes a day for a minimum of the preceding 5 years. The study material, venous blood, was taken fasting from the basilic vein of the study group members at 4 time points: prior to giving up smoking, as well as after the 1st, 2nd, and 3rd months of tobacco abstinence. Throughout the experimental period, the study group members were monitored in their workplace for maintaining tobacco abstinence and received incentive payments.

 The study also included two control groups composed of 35 subjects:

1. healthy volunteers (without COPD), who are nonsmoking either at the time of the study or ever before, not exposed to smoke at home/work, and aged from 18 to 73 (mean age = 44.7 ± 15.1 years) as Control I,
2. cigarette smokers (as in the study group, smoking at least 10 cigarettes a day for a minimum of the preceding 5 years) with COPD, who had not given up smoking, aged 29 to 74 (mean age = 49.8 ± 11.1 years) as Control II.

Subjects with other health problems characterized by a proven oxidant-antioxidant imbalance were excluded from the study.

 Blood sampling from healthy volunteers (Control I) was performed one time, while in the case of the COPD patients who had not given up smoking, blood samples were obtained 4 times at 1-month intervals. As in the case of the study group, blood was taken fasting from the basilic vein.

 All study subjects were asked not to change their diet throughout the experimental period. Patients from the study group and Control II were assigned GOLD Category A of COPD with a low acuteness of the disease and mild symptoms. In 33 patients from the study group, a mild degree of obturation was determined (Forced Expiratory Volume in 1 second—FEV_1_ > 80% predicted value; the FEV_1_/FVC ratio was below the lower limit of normal, where FVC is the Forced Vital Capacity of the lungs), while in 40 subjects, the degree was moderate (50% predicted value < FEV_1_ < 80% predicted value; the FEV_1_/FVC ratio was below the lower limit of normal). Among the patients from Control II, 17 of them presented a mild degree of obturation development, while in 18 of them, the degree was moderate (the FEV_1_/FVC ratio below the lower limit of normal in all cases). The patients from both groups were not receiving any regular pharmacological treatment. Sporadically, depending on the needs, antihistamines, inhaled steroids, or *β*-mimetics, as well as mucolytic medications, were administered.

Venous blood for full blood analysis was collected into tubes containing potassium versenate (K_2_EDTA, ethylenediaminetetraacetic acid dipotassium salt). The activity of SOD, CAT, and GPx was determined in erythrocytes. The levels of TBARS and CDs were determined in erythrocytes and blood plasma. Blood plasma was also assessed for the concentration of low molecular weight antioxidants and vitamins E and A. The diagnosis of COPD was made based on anamnesis, physical examination, and spirometry (KoKo Legend Spirometer, Ferraris Systems), whose aim was to confirm obstructive-type disorders. 

### 2.1. Assay of Lipid Peroxidation Products in Blood Plasma and Erythrocytes

CD levels were determined following Sergent et al. [20]. Conjugated dienes are formed in the process of lipid peroxidation, as a result of a reconfiguration of double bonds following the detachment of a hydrogen atom from a polyunsaturated fatty acid molecule and yield a characteristic absorbance peak at *λ* = 233 nm. The samples obtained once the hemolyzed erythrocytes or blood plasma centrifuged with chloroform, evaporated in nitrogen atmosphere, and dissolved in cyclohexane, and their absorbance was measured. CD concentration was expressed in absorbance units per mL plasma (Abs./mL) and absorbance units per gram Hb (Abs./g Hb). The levels of thiobarbituric acid-reactive substances (TBARSs) were determined following the Buege and Aust [21] method as modified by Esterbauer and Cheesman [22]. The method involves creation of a coloured complex between lipid peroxidation products and thiobarbituric acid (TBA) at the temperature of 100°C and in acidic environment. The maximum absorption of that complex occurs at a wavelength of 532 nm. To prevent the occurrence of peroxidation products during the reaction, 0.01% solution of 3,5-dibutyl-4-hydroxytoluene (BHT) was added to the reaction tubes. The main product of lipid peroxidation reacting with TBA is malondialdehyde (MDA), and, therefore, the levels of TBARS in plasma were expressed as nmol MDA/mL, while in the erythrocytes, as nmol MDA/g Hb. 

### 2.2. Assay of Antioxidant Enzymes Activity in Erythrocytes

SOD activity was determined using the Misra and Fridovich method [23]. This procedure is based on SOD impeding the reaction of autooxidation of adrenaline to adrenochrome in an alkaline environment. Absorbance measurements were taken at *λ* = 480 nm, and the SOD activity was expressed in U/g Hb. The Beers and Sizer [24] method was used to assay the CAT activity. This method is based on the measurement of absorbance decrease, measured at a wavelength of 240 nm, as hydrogen peroxide is decomposed by CAT. The CAT activity was expressed in IU/g Hb. GPx activity was determined following Paglia and Valentine [25], that is, using the oxidation of reduced glutathione (GSH) by H_2_O_2_, catalysed by GPx. Oxidized glutathione is then reduced by exogenous glutathione reductase. This causes nicotinamide adenine dinucleotide phosphate (NADPH), a coenzyme in this reaction, to be oxidized into NADP^+^ which induces a change in the absorbance at *λ* = 340 nm. The activity of GPx was expressed in U/g Hb. 

### 2.3. Assay of Antioxidant Vitamin Level in Blood Plasma

The levels of vitamins A and E were determined using a high performance liquid chromatography (HPLC) system [26]. As an internal reference, retinyl acetate was used for vitamin A- and *α*-tocopheryl acetate was used for vitamin E. Acetonitrile was used as a protein-denaturing agent. The mix of vitamins was separated using the Kinetex 2.6 *μ*m C18 75 × 4.6 mm chromatography column with the mobile phase containing acetonitrile : methanol (at the 95 : 5 ratio) at the flow rate of 2 mL/min. The vitamins were detected using a UV/Vis detector at *λ* = 325 nm for vitamin A and *λ* = 295 nm for vitamin E. Their concentrations were determined using the WorkStation Polaris software and expressed in *μ*g/L.

### 2.4. Statistical Analysis

Statistical analysis was conducted using the ANOVA test with post hoc analysis (Tukey's range test) (*STATISTICA v.* 9.1). A hypothesis of the equality of two means was tested. Differences at a significance level *P* < 0.05 were considered as statistically significant. Dependencies between the analysed parameters were assessed using correlation matrices. A statistical hypothesis of the significance of the correlation coefficients (*r*) was tested.

## 3. Results

### 3.1. Spirometric Parameters and the Response to Smoking Cessation

FEV_1_ and FVC expressed as the percentage of the predicted values and the FEV_1_/FVC ratio were very similar in both groups of patients with COPD before smoking cessation/at the beginning of the study, while being significantly lower than the values obtained in Control I (healthy nonsmokers) by approx. 25% (*P* < 0.001), 13% (*P* < 0.001), and 27% (*P* < 0.001), respectively (Table 2). The values of FEV_1_, FVC, and the FEV_1_/FVC ratio obtained at every other stage of the study were also significantly lower in the COPD patients (study group and Control II) than in Control I. Tobacco abstinence did not significantly affect the aforementioned parameters of lung function. Similarly, in the COPD patients who had not given up smoking, FEV_1_, FVC, and the FEV_1_/FVC ratio did not change in a statistically significant manner at any stage of the study.

### 3.2. Lipid Peroxidation Biomarkers and the Response to Smoking Cessation

In the study group patients, before smoking cessation, the level of lipid peroxidation products both in blood plasma and in erythrocytes was significantly higher than in the healthy representatives of Control I (Table 3). The plasma CD level in those patients was over three times higher (*P* < 0.001) while the erythrocyte CD level was approx. 6.5 times higher (*P* < 0.001) than in Control I. The TBARS concentration in the patients was almost twice as high (*P* < 0.001) in blood plasma and 1.5 times higher in the erythrocytes (*P* < 0.01). After the 3rd month of tobacco abstinence, the plasma and erythrocyte CD and TBARS levels were significantly lower by 46% (*P* < 0.001), 71% (*P* < 0.001), 42% (*P* < 0.001), and 31% (*P* < 0.01), respectively, as compared with the values observed before smoking cessation. The erythrocyte CD level in the patients with COPD was significantly lower already after the 1st and 2nd months of tobacco abstinence by 33% (*P* < 0.001) and 51% (*P* < 0.001), respectively, as compared with the values observed before smoking cessation. The concentration of lipid peroxidation products measured in the COPD patients after the 3rd month of tobacco abstinence did not present a statistically significant difference from the values obtained in Control I, nevertheless, it was slightly higher. The TBARS level in erythrocytes decreased to the value that was not significantly different from those observed in Control I already after the 2nd month of tobacco abstinence.

The plasma CD level in the COPD patients who had not given up smoking (Control II) at the beginning and after the 1st, 2nd, and 3rd months of the study was over three times higher (*P* < 0.001) while the erythrocyte CD level was approx. 7-8 times higher (*P* < 0.001) than in Control I (Table 3). The TBARS level in the patients from Control II was approx. twice as high (*P* < 0.001) in blood plasma and approx. 1.5 times higher (*P* < 0.001) in erythrocytes than in Control I. The comparison of the concentration of lipid peroxidation products observed in both groups of patients (study group and Control II) revealed statistically significant differences between the levels of CDs and TBARS measured in the study group after the 3rd month of tobacco abstinence and the levels of those products in Control II at all stages of the study. After the 3rd month of tobacco abstinence, the level of CDs was lower by 51% (*P* < 0.001) in blood plasma and by 75% (*P* < 0.001) in erythrocytes, as compared with the values obtained after the 3rd month of the study in Control II. At the same time, the level of TBARS after the 3rd month of tobacco abstinence was lower by 46% (*P* < 0.001) in blood plasma and by 30% (*P* < 0.05) in erythrocytes, as compared with the values obtained in the 3rd month of the study in Control II. As for the erythrocyte CD level, statistically significant differences were observed already after the 1st and 2nd months of tobacco abstinence, as compared with the results obtained at the appropriate study stages in Control II. The concentration of this early product of lipid peroxidation in the study group was lower by 36% (*P* < 0.001) after the 1st month and by 58% (*P* < 0.001) after the 2nd months of tobacco abstinence, as compared with the concentrations measured after the 1st and 2nd month of tobacco abstinence in the patients from Control II, respectively.

### 3.3. Antioxidant Enzymes Activity and the Response to Smoking Cessation

In the study group before smoking cessation, the erythrocyte activity of SOD was higher by 40%  (*P* < 0.05) while that of GPx was lower by 41% (*P* < 0.001), as compared with the values obtained in the representatives of Control I (Table 4). After the 3rd month of tobacco abstinence, the SOD activity was decreased by 29% (*P* < 0.01) while the GPx activity increased by 35% (*P* < 0.01), as compared with the values obtained before smoking cessation. The GPx activity measured at that stage of the study still remained 20% lower than that of Control I (*P* < 0.05), whereas the appropriate SOD activity became similar to that observed in the erythrocytes of the representatives of Control I. The SOD activity determined after the 3rd month of tobacco abstinence was also lower by approx. 26% than that obtained after the 1st (*P* < 0.01) and 2nd months of tobacco abstinence (*P* < 0.05). On the other hand, the GPx activity measured after the 3rd month of tobacco abstinence was 35% higher (*P* < 0.01) than that determined after the 1st month of tobacco abstinence. No statistically significant changes were found in the activity of CAT in the erythrocytes of the COPD patients who had given up smoking. The comparison of the erythrocyte activity of SOD, GPx, and CAT in both groups of patients, based on the results obtained before smoking cessation/at the beginning of the study, did not reveal any statistically significant differences.

In the COPD patients who had not given up smoking (Control II), the activity of SOD at the beginning of the study was 44% higher (*P* < 0.01) than in the healthy nonsmokers from Control I, whereas the activity of GPx was 36% lower (*P* < 0.001) (Table 4). During the subsequent stages of the study, a further statistically insignificant tendency of the SOD activity to increase (apart from the activity measured after the 1st month) and the GPx activity to decrease (apart from the unchanged activity measured after the 3rd month) was observed. After the 1st, 2nd, and 3rd months of the study, the activity of SOD in Control II was higher than in Control I by 42% (*P* < 0.01), 55% (*P* < 0.001), and 57% (*P* < 0.001), respectively, while the activity of GPx was lower by 38% (*P* < 0.001), 43% (*P* < 0.001), and 43% (*P* < 0.001), respectively. No statistically significant changes in the CAT activity were observed throughout the study in the patients from Control II.

### 3.4. Antioxidant Vitamins Level and the Response to Smoking Cessation

Neither significant differences in the plasma level of the small-molecule antioxidants, vitamins A and E, were observed between the patients from the study group and the healthy subjects from Control I (Table 4). However, the concentrations of both vitamins in the former group were insignificantly lower than in the latter group. Before smoking cessation, the levels of vitamins A and E were approx. 15% lower (*P* > 0.05) in the study group. After the 1st, 2nd, and 3rd months of tobacco abstinence, an insignificant tendency of the concentrations of both vitamins to increase was detected.

Neither significant differences in the plasma level of vitamin A were established in the healthy subjects (Control I) and the patients (Control II), even though at every stage of the study, the values obtained in Control II were insignificantly lower than those in Control I (Table 4). Moreover, the concentration of vitamin E after the 2nd and 3rd months of the study in Control II was lower by 36% (*P* < 0.001) and 37% (*P* < 0.001), respectively, as compared with Control I. The comparison of the plasma levels of vitamin E in the patients (study group and Control II) revealed that higher values were measured after the 1st, 2nd, and 3rd months of tobacco abstinence, as compared with the results obtained in Control II after the 2nd and 3rd months of the study. After the 1st month of tobacco abstinence, the levels of vitamin E were higher by 49% (*P* < 0.01) and 52% (*P* < 0.001), respectively, as compared with the values observed after the 2nd and 3rd months of the study, and after the 2nd month of tobacco abstinence, the levels were higher by 51% (*P* < 0.001) and 54% (*P* < 0.001), respectively, while after the 3rd month of tobacco abstinence, the levels were higher by 54% (*P* < 0.001) and 57% (*P* < 0.001), respectively. No statistically significant differences were established in the comparison of the plasma levels of vitamin A in the study group and Control II.

### 3.5. Correlation Analysis

A series of statistically significant correlations between the determined parameters was found. In the study group patients before smoking cessation, positive correlations were found between the activity of SOD and CAT (*r* = 0.44,  *P* < 0.001), as well as between the CD level in blood plasma and in erythrocytes (*r* = 0.64, *P* < 0.001), whereas a negative correlation was found between FEV_1_ and the level of vitamin E (*r* = −0, 26, *P* < 0.05). After the 1st month of tobacco abstinence, a statistically significant positive correlation was observed between FEV_1_ and FVC (*r* = 0.43, *P* = 0.001), while after the 2nd month of tobacco abstinence, such a correlation was observed between the activity of SOD and CAT (*r* = 0.55, *P* < 0.001). After the 3rd month of tobacco abstinence, a positive correlation was found between the activity of SOD and CAT (*r* = 0.50, *P* < 0.001), whereas negative correlations were found between the activity of CAT and the plasma level of TBARS (*r* = −0.48, *P* < 0.01), as well as between the activity of CAT and the level of vitamin A (*r* = −0.49, *P* < 0.01).

In Control I, a positive correlation was observed between the activity of CAT and the erythrocyte level of TBARS (*r* = 0.63,  *P* < 0.001). In Control II, at the beginning of the study, a negative correlation was established between the plasma level of TBARS and the FEV1/FVC ratio (*r* = −0.56, *P* < 0.001). After the 1st month of the study in control II, a positive correlation was observed between the plasma CD level and FVC (*r* = 0.48, *P* < 0.01), whereas after the 2nd month, such a correlation was noted between the FEV_1_/FVC ratio and FEV_1_ (*r* = 0.34, *P* < 0.05). After the 3rd month of the study, a negative correlation was found between the activity of SOD and FEV_1_ (*r* = −0.53, *P* = 0.001).

## 4. Discussion

 The higher levels of CDs and TBARS, observed in the erythrocytes and blood plasma in both groups of COPD patients at the beginning of the study/before smoking cessation, as compared with the healthy nonsmokers from Control I, is a sign of an increased peroxidation of lipids forming the membranes of erythrocytes and those present in blood plasma. Increased TBARS levels in the erythrocytes of COPD patients were also detected by Lee [27]. Likewise, increased peroxidation of lipids and oxidation of proteins of the erythrocytes and platelets from patients with COPD and asthma were confirmed by de Castro et al. [28]. A higher concentration of MDA in the blood plasma of COPD patients, as compared with that found in healthy subjects, was found by Cristóvão et al. [29]. Folchini et al. [30] observed statistically significant differences between the blood TBARS levels in patients with COPD depending on the gravity of the disease. The authors proved that patients with grade 4 COPD have higher levels of those peroxidation products than patients with grade 1 COPD.

 The increased ROS generation in COPD patients is also confirmed by the changes in the activity of antioxidant enzymes, revealed by the results obtained in the above mentioned study. The activity of SOD in the erythrocytes of the COPD patients was higher than in the healthy representatives of Control I. In contrast, the activity of GPx was significantly lower, while that of CAT did not differ in a significant manner from the value obtained in Control I.

 The lower GPx activity in the COPD patients, as compared with that of Control I, with the concurrently higher erythrocyte levels of TBARS confirm the presence of disturbances in the functioning of the antioxidant enzyme barrier. The decrease in the activity of the enzyme may be a consequence of the decrease in GSH levels in COPD patients, which was confirmed by Çalikoğlu et al. [31], or may be related with the levels of selenium. Lower selenium concentrations in the blood plasma of COPD patients, either present or former smokers, as compared with the values obtained in healthy subjects, were observed by Santos et al. [32].

 Changes in the activity of antioxidant enzymes in the erythrocytes of COPD patients, similar to those presented in our study, were observed by Nadeem et al. [33]. The authors detected a higher activity of SOD, a lower activity of GPx, and a similar activity of CAT, as compared with those found in the control group (healthy nonsmokers). A lower activity of GPx in erythrocytes occurred both in patients with moderate and severe forms of COPD, as compared with the activity established in healthy subjects [34]. A higher activity of SOD, as compared with that in healthy subjects, was revealed by Hanta et al. [35] in those COPD patients, in whom disease exacerbation occurred. The authors claimed that the increased activity of the enzyme may be a result of the induction of the SOD gene expression by oxidants. Waseem et al. [36] determined the activity of GPx, SOD, and CAT, as well as the plasma levels of MDA in smokers with COPD and in healthy nonsmokers as a control. In the patients, the authors revealed a significantly higher concentration of MDA and a lower activity of all analysed antioxidant enzymes. Apparently, the discrepancies between the changes in the activities of the analysed antioxidant enzymes found by other authors and those published in this paper may be a result of a different severity of COPD in the study subjects.

 The concentrations of the assessed antioxidant vitamins presented in this paper prove the increased usage of those substances in the patients with COPD. The decreased levels of vitamins E and C in the blood plasma of COPD patients, as compared with those measured in healthy subjects, were also detected by Rai and Phadke [37]. In healthy smokers, decreased levels of vitamins C and E, as well as *β*-carotene, were observed by Zhou et al. [38]. The authors compared the concentration of those vitamins in smokers and healthy nonsmokers of a similar age. Lin et al. [39] also found lower levels of vitamins A, E, and C, as well as *α* and *β*-carotene, and carotenoids in general, in COPD patients as compared with healthy subjects.

 In the presented study, a tendency of the concentration of vitamins A and E to increase was observed in the group of COPD patients who had given up smoking, which may confirm that smaller quantities of these compounds are used due to a lower ROS generation, particularly since in Control II, a group of COPD patients who had not given up smoking, the levels of both vitamins exhibited a tendency to decrease. Therefore, it seems that cigarette smoke, as a direct and indirect source of ROS, may be responsible for the observed changes in the concentration of antioxidant vitamins.

 The positive effect of smoking cessation on the oxidant-antioxidant balance in the peripheral blood of COPD patients is also supported by the changes in the concentrations of lipid peroxidation products and the activity of antioxidant enzymes described in this paper. After the 3rd month of tobacco abstinence, a statistically significant decrease in the levels of lipid peroxidation products was demonstrated, as compared with the levels detected before smoking cessation. A decrease in the erythrocyte CD level in the COPD patients was observed already after the 1st month of tobacco abstinence. CDs are the so-called early products of the lipid peroxidation process, whose assessment, according to Jenkins et al. [40], may prove the presence of oxidative stress. The decreased level of CDs may therefore be a sign of an ongoing restoration of the oxidant-antioxidant balance. The erythrocyte activity of SOD after the 3rd month of tobacco abstinence decreased significantly, while that of GPx exhibited a statistically insignificant increase, as compared with the values measured before smoking cessation. The activity of SOD after the 3rd month of tobacco abstinence became similar to that observed in Control I, while the activity of GPx at that stage of the study remained significantly lower that in Control I. In the patients who had not given up smoking, no statistically significant differences in the changes of the activity of the assessed enzymes were found at any stage of the study.

 Other authors also indicate a possibility of restoring the oxidant-antioxidant balance in COPD patients after smoking cessation or treatment. Hanta et al. [35] observed a lower activity of SOD in the erythrocytes of the COPD patients suffering from either a stable disease or an exacerbated disease, who had not smoked for at least a year, as compared with present smokers. Sahin et al. [41] demonstrated that, at the same time, the activity of GPx in the erythrocytes of smoking COPD patients was lower than in those patients who had given up smoking. In contrast, the plasma levels of MDA were higher in the smoking patients than in the former smokers. Conversely, Santos et al. [32] did not observe any differences in the activity of SOD and GPx in the erythrocytes of present and former smokers. A lack of statistically significant differences in the plasma levels of MDA in currently and formerly smoking COPD patients was found by Karadag et al. [42].

 In our study presented in this paper, no changes in the assessed spirometric parameters, FEV_1_, FVC, and the FEV_1_/FVC ratio, were observed. Apparently, the 3-month period of tobacco abstinence is too short to permit improvement in the lung function. Similar conclusions were drawn by Louhelainen et al. [43] who performed three spirometric tests per subject: before smoking cessation, after the 1st and 3rd months of tobacco abstinence, and did not reveal any statistically significant changes in FVC, FEV_1_, and the FEV_1_/FVC ratio. 

 In contrast, Yang [44] proved that giving up smoking protects from further decreases in such parameters as FVC and FEV_1_. However, the study was conducted in smokers not suffering from COPD. Scanlon et al. [19], by conducting tests in 926 former smokers with mild or moderate COPD, demonstrated a 2% (47 mL) improvement in the FEV_1_ value after one year of tobacco abstinence. In the subjects who continued smoking throughout the study period of one year, the FEV_1_ parameter decreased. The level of oxidative stress decreases as the tobacco abstinence period becomes longer, but the time necessary for the improvement to occur remains unknown [43]. 

## 5. Conclusions

 The obtained results prove that COPD is accompanied by oxidative stress. A three-month period of tobacco abstinence facilitated restoring the oxidant-antioxidant balance systemically, but it did not affect spirometric parameters. Determining the concentrations of lipid peroxidation products, enzyme activity and the levels of antioxidant vitamins in the peripheral blood may be employed in the diagnostics of COPD and in monitoring tobacco abstinence.

## Figures and Tables

**Table 1:** Patient characteristics.

	Healthy subjects, nonsmoking—control I	COPD patients—control II	COPD patients—study group
Number of subjects	35	35	73
Age (years)	44.7 ± 15.1	49.8 ± 11.1	48.9 ± 11.7
Sex (F/M)	16/19	14/21	33/40
Smoking period (years)	—	31.8 ± 11.1	30.1 ± 13.1
No. of packs/year	—	299.0 ± 66.7	288.7 ± 78.6

COPD: chronic obstructive pulmonary disease.

Data expressed as mean $\bar{x}\pm \text{SD}$.

**Table 2:** Spirometric parameters in the COPD patients who had given up smoking and in the representatives of the control groups: COPD patients who had not given up smoking and healthy subjects.

Group	FEV_1_ (% predicted value)	Parameters FVC (% predicted value)	FEV_1_/FVC (%)
Control I (healthy nonsmokers)	97.7 ± 14.0	109.1 ± 13.3	84.7 ± 5.7

COPD patients who had not given up smoking (Control II)			
At the beginning of the study	73.2 ± 19.5***	94.6 ± 15.6***	62.3 ± 6.6***
After the 1st month of the study	73.3 ± 20.4***	93.5 ± 17.0***	61.9 ± 5.4***
After the 2nd month of the study	73.1 ± 14.7***	94.0 ± 18.5***	61.5 ± 7.5***
After the 3rd month of the study	73.1 ± 17.1***	93.8 ± 22.8***	61.2 ± 5.9***

COPD patients who had given up smoking (study group)			
Before smoking cessation	73.5 ± 17.8***	94.6 ± 17.5***	61.9 ± 7.6***
After the 1st month of tobacco abstinence	74.0 ± 25.5***	94.1 ± 16.3***	61.2 ± 6.1***
After the 2nd month of tobacco abstinence	74.3 ± 23.6***	94.3 ± 14.6***	61.4 ± 6.4***
After the 3rd month of tobacco abstinence	74.8 ± 24.6***	94.5 ± 14.6*	62.1 ± 6.3***

COPD: chronic obstructive pulmonary disease; FEV_1_: forced expiratory volume in 1 second; FVC: forced vital capacity; FEV_1_/FVC: forced expiratory volume in 1 second/forced vital capacity ratio.

Data expressed as mean $\bar{x}\pm \text{SD}$; *P < 0.05; ***P < 0.001 compared with Control I.

**Table 3:** Levels of lipid peroxidation products in the COPD patients who had given up smoking and in the representatives of the control groups: COPD patients who had not given up smoking and healthy subjects.

Group	Parameters			
	CD_plasma_ (10^−2^ Abs./mL)	CD_eryth._ (10^−1^ Abs./g Hb)	TBARS_plasma_ (10^−1^ nmol MDA/mL)	TBARS_eryth._ (nmol MDA/g Hb)
Control I (healthy nonsmokers)	4.1 ± 1.5	1.4 ± 0.2	2.8 ± 0.3	25.0 ± 6.5

COPD patients who had not given up smoking (Control II)				
At the beginning of the study	13.8 ± 5.8***	9.5 ± 3.3***	5.1 ± 1.5***	37.1 ± 17.9**
After the 1st month of the study	14.5 ± 5.5***	9.7 ± 2.7***	5.2 ± 1.5***	37.7 ± 15.1**
After the 2nd month of the study	14.9 ± 3.9***	11.0 ± 4.7***	5.3 ± 1.5***	37.5 ± 11.6**
After the 3rd month of the study	14.9 ± 3.5***	10.9 ± 2.8***	5.7 ± 1.6***	37.3 ± 12.9**

COPD patients who had given up smoking (study group)				
Before smoking cessation	13.5 ± 5.8***	9.3 ± 4.3***	5.3 ± 1.8***	37.8 ± 17.3**
After the 1st month of tobacco abstinence	13.4 ± 5.4***	6.2 ± 1.7^∗∗∗aaabbbcccdddeee^	5.3 ± 1.7***	38.1 ± 16.2**
After the 2nd month of tobacco abstinence	11.8 ± 3.3***	4.6 ± 2.2^∗∗∗aaabbbcccdddeee^	4.9 ± 1.5***	33.0 ± 11.4
After the 3rd month of tobacco abstinence	7.3 ± 2.6^aaabbbcccdddeeefffggg^	2.7 ± 1.1^aaabbbcccdddeeefffg^	3.1 ± 0.7^aaabbbcccdddeeefffggg^	26.1 ± 9.3^abcdeeff^

COPD: chronic obstructive pulmonary disease; CDs: conjugated dienes; TBARS: thiobarbituric acid-reactive substances.

Data expressed as mean $\bar{x}\pm \text{SD}$.

Statistically significant differences versus Control I: **P < 0.01, ***P < 0.001; versus Control II at the beginning of the study: ^a^ *P* < 0.05, ^aaa^ *P* < 0.001; versus Control II after the 1st month: ^b^ *P* < 0.05, ^bbb^ *P* < 0.001; versus Control II after the 2nd month: ^c^ *P* < 0.05, ^ccc^ *P* < 0.001; versus Control II after the 3rd month: ^d^ *P* < 0.05, ^ddd^ *P* < 0.001; versus the study group before smoking cessation: ^ee^ *P* < 0.01, ^eee^ *P* < 0.001; versus the study group after the 1st month of tobacco abstinence: ^ff^ *P* < 0.01, ^fff^ *P* < 0.001; versus the study group after the 2nd month of tobacco abstinence: ^g^ *P* < 0.05, ^ggg^ *P* < 0.001.

**Table 4:** Enzyme activity and the levels of antioxidant vitamins in the COPD patients who had given up smoking and in the representatives of the control groups: COPD patients who had not given up smoking and healthy subjects.

Group	Parameters				
	SOD (U/g Hb)	GPx (U/g Hb)	CAT (10^4^ IU/g Hb)	Vit. A (*μ*g/L)	Vit. E (10^2^ *μ*g/L)
Control I (healthy nonsmokers)	1114.7 ± 176.9	13.8 ± 4.6	57.6 ± 9.9	325.3 ± 98.2	30.2 ± 6.4

COPD patients who had not given up smoking (Control II)					
At the beginning of the study	1606.2 ± 501.0**	8.8 ± 3.1***	53.9 ± 13.2	279.0 ± 89.1	25.0 ± 9.4
After the 1st month of the study	1585.8 ± 472.7**	8.6 ± 4.1***	53.2 ± 12.1	277.3 ± 96.0	24.6 ± 8.1
After the 2nd month of the study	1723.2 ± 439.4***	7.8 ± 3.3***	51.3 ± 15.4	273.7 ± 93.3	19.3 ± 8.4***
After the 3rd month of the study	1747.5 ± 478.8***	7.8 ± 3.0***	50.3 ± 19.8	272.0 ± 86.8	18.9 ± 6.9***

COPD patients who had given up smoking (study group)					
Before smoking cessation	1564.3 ± 581.1*	8.2 ± 3.1***	53.7 ± 12.9	275.8 ± 88.4	25.8 ± 8.0
After the 1st month of tobacco abstinence	1511.0 ± 700.8*	8.2 ± 3.8***	54.5 ± 13.1	295.0 ± 100.9	28.8 ± 14.0^ccddd^
After the 2nd month of tobacco abstinence	1506.7 ± 630.9	9.2 ± 3.2***	55.1 ± 14.9	313.7 ± 109.9	29.2 ± 11.6^cccddd^
After the 3rd month of tobacco abstinence	1117.4 ± 343.6^aabbcccdddeeffg^	11.1 ± 3.7^∗ccddeeff^	56.3 ± 14.7	315.2 ± 106.2	29.7 ± 9.9^cccddd ^

COPD: chronic obstructive pulmonary disease; SOD: superoxide dismutase; GPx: glutathione peroxidase; CAT: catalase; Vit. E, A: vitamins E, A.

Data expressed as mean $\bar{x}\pm \text{SD}$.

Statistically significant differences versus Control I: *P < 0.05, **P < 0.01, and ***P < 0.001; versus

Control II at the beginning of the study: ^aa^ *P* < 0.01; versus Control II after the 1st month: ^bb^ *P* < 0.01; versus Control II after the 2nd month: ^cc^ *P* < 0.01, ^ccc^ *P* < 0.001; versus Control II after the 3rd month: ^dd^ *P* < 0.01, ^ ddd^ *P* < 0.001; versus the study group before smoking cessation: ^ee^ *P* < 0.01; versus the study group after the 1st month of tobacco abstinence: ^ff^ *P* < 0.01; versus the study group after the 2nd month of tobacco abstinence: ^g^ *P* < 0.05.

## References

[1] Devereux G (2006). ABC of chronic obstructive pulmonary disease: definition, epidemiology, and risk factors. *British Medical Journal*.

[2] Panzner P (2002). Cytokines in chronic obstructive pulmonary disease and chronic bronchitis. *Alergia Astma Immunologia*.

[3] Barnes PJ, Shapiro SD, Pauwels RA (2003). Chronic obstructive pulmonary disease: molecular and cellular mechanisms. *European Respiratory Journal*.

[4] Wada H, Takizawa H (2013). Future treatment for COPD: targeting oxidative stress and its related signal. *Recent Patents on Inflammation & Allergy Drug Discovery*.

[5] Tam A, Sin DD (2012). Pathobiologic mechanisms of chronic obstructive pulmonary disease. *The Medical Clinics of North America*.

[6] Miglino N, Roth M, Tamm M, Borger P (2012). Asthma and COPD—the C/EBP connection. *The Open Respiratory Medicine Journal*.

[7] MacNee W, Rahman I (2000). Is oxidative stress central to the pathogenesis of chronic obstructive pulmonary disease?. *Trends in Molecular Medicine*.

[8] Mateos F, Brock JH, Pérez-Arellano JL (1998). Iron metabolism in the lower respiratory tract. *Thorax*.

[9] Rahman I, Skwarska E, MacNee W (1997). Attenuation of oxidant/antioxidant imbalance during treatment of exacerbations of chronic obstructive pulmonary disease. *Thorax*.

[10] Komaki Y, Sugiura H, Koarai A (2005). Cytokine-mediated xanthine oxidase upregulation in chronic obstructive pulmonary disease’s airways. *Pulmonary Pharmacology and Therapeutics*.

[11] Richter C, Gogvadze V, Laffranchi R (1995). Oxidants in mitochondria: from physiology to diseases. *Biochimica et Biophysica Acta*.

[12] Pratt DA, Tallman KA, Porter NA (2011). Free radical oxidation of polyunsaturated lipids: new mechanistic insights and the development of peroxyl radical clocks. *Accounts of Chemical Research*.

[13] Favier A (1997). The oxidative stress: Interest of its monitoring in clinical chemistry and problems of the choice of an appropriate parameter. *Annales de Biologie Clinique*.

[14] Woźniak A (2003). Signs of oxidative stress after exercise. *Biology of Sport*.

[15] Michel F, Bonnefont-Rousselot D, Mas E, Drai J, Thérond P (2008). Biomarkers of lipid peroxidation: analytical aspects. *Annales de Biologie Clinique*.

[16] Cantin AM (2010). Cellular response to cigarette smoke and oxidants: adapting to survive. *Proceedings of the American Thoracic Society*.

[17] Mak JCW (2008). Pathogenesis of COPD. Part II. Oxidative-antioxidative imbalance. *International Journal of Tuberculosis and Lung Disease*.

[18] Hasnain BI, Mooradian AD (2004). Recent trials of antioxidant therapy: what should we be telling our patients?. *Cleveland Clinic Journal of Medicine*.

[19] Scanlon PD, Connett JE, Waller LA (2000). Smoking cessation and lung function in mild-to-moderate chronic obstructive pulmonary disease: the lung health study. *American Journal of Respiratory and Critical Care Medicine*.

[20] Sergent O, Morel I, Cogrel P (1993). Simultaneous measurements of conjugated dienes and free malondialdehyde, used as a micromethod for the evaluation of lipid peroxidation in rat hepatocyte cultures. *Chemistry and Physics of Lipids*.

[21] Buege JA, Aust SD (1978). Microsomal lipid peroxidation. *Methods in Enzymology*.

[22] Esterbauer H, Cheeseman KH (1990). Determination of aldehydic lipid peroxidation products: malonaldehyde and 4-hydroxynonenal. *Methods in Enzymology*.

[23] Misra HP, Fridovich I (1972). The role of superoxide anion in the autoxidation of epinephrine and a simple assay for superoxide dismutase. *The Journal of Biological Chemistry*.

[24] Beers RF, Sizer IW (1952). A spectrophotometric method for measuring the breakdown of hydrogen peroxide by catalase. *The Journal of biological chemistry*.

[25] Paglia DE, Valentine WN (1967). Studies on the quantitative and qualitative characterization of erythrocyte glutathione peroxidase. *The Journal of Laboratory and Clinical Medicine*.

[26] Wieliński S, Olszanowski A (1999). Practical optimization of solvent selectivity using gradient elution for rapid selection and mixture-design statistical technique for the separation of fat-soluble vitamins. *Journal of Liquid Chromatography and Related Technologies*.

[27] Lee S-I (1997). The level of antioxidant enzymes in red blood cells of patients with chronic obstructive pulmonary disease. *Tuberculosis and Respiratory Diseases*.

[28] De Castro J, Hernández-Hernández A, Rodríguez MC, Sardina JL, Llanillo M, Sánchez-Yagüe J (2007). Comparison of changes in erythrocyte and platelet phospholipid and fatty acid composition and protein oxidation in chronic obstructive pulmonary disease and asthma. *Platelets*.

[29] Cristóvão C, Cristóvão L, Nogueira F, Bicho M (2013). Evaluation of the oxidant and antioxidant balance in the pathogenesis of chronic obstructive pulmonary disease. *Revista Portuguesa De Pneumologia*.

[30] Folchini F, Nonato NL, Feofiloff E, D’Almeida V, Nascimento O, Jardim JR (2011). Association of oxidative stress markers and C-reactive protein with multidimensional indexes in COPD. *Chronic Respiratory Disease*.

[31] Çalikoğlu M, Ünlü A, Tamer L, Ercan B, Buğdayci R, Atik U (2002). The levels of serum vitamin C, malonyldialdehyde and erythrocyte reduced glutathione in chronic obstructive pulmonary disease and in healthy smokers. *Clinical Chemistry and Laboratory Medicine*.

[32] Santos MC, Oliveira AL, Viegas-Crespo AM (2004). Systemic markers of the redox balance in chronic obstructive pulmonary disease. *Biomarkers*.

[33] Nadeem A, Raj HG, Chhabra SK (2005). Increased oxidative stress and altered levels of antioxidants in chronic obstructive pulmonary disease. *Inflammation*.

[34] Torres-Ramos YD, Montoya-Estrada A, Guzman-Grenfell AM (2011). Urban PM2.5 induces ROS generation and RBC damage in COPD patients. *Frontiers in Bioscience*.

[35] Hanta I, Kocabas A, Canacankatan N, Kuleci S, Seydaoglu G (2006). Oxidant-antioxidant balance in patients with COPD. *Lung*.

[36] Waseem SMA, Hossain MM, Islam N, Ahmad Z (2012). Comparative study of pulmonary functions and oxidative stress in smokers and non-smokers. *Indian Journal of Physiology and Pharmacology*.

[37] Rai RR, Phadke MS (2006). Plasma oxidant-antioxidant status in different respiratory disorders. *Indian Journal of Clinical Biochemistry*.

[38] Zhou J, Guo F, Qian Z (1997). Effects of cigarette smoking on antioxidant vitamin and activities of antioxidases. *Zhonghua Yu Fang Yi Xue Za Zhi*.

[39] Lin Y-C, Wu T-C, Chen P-Y, Hsieh L-Y, Yeh S-L (2010). Comparison of plasma and intake levels of antioxidant nutrients in patients with chronic obstructive pulmonary disease and healthy people in Taiwan: a case-control study. *Asia Pacific Journal of Clinical Nutrition*.

[40] Jenkins RR, Krause K, Schofield LS (1993). Influence of exercise on clearance of oxidant stress products and loosely bound iron. *Medicine and Science in Sports and Exercise*.

[41] Sahin Ü, Ünlü M, Özgüner F, Sütcü R, Akkaya A, Delibas N (2001). Lipid peroxidation and glutathione peroxidase activity in chronic obstructive pulmonary disease exacerbation: prognostic value of malondialdehyde. *Journal of Basic and Clinical Physiology and Pharmacology*.

[42] Karadag F, Karul AB, Cildag O, Altun C, Gurgey O (2004). Determinants of BMI in patients with COPD. *Respirology*.

[43] Louhelainen N, Rytilä P, Haahtela T, Kinnula VL, Djukanović R (2009). Persistence of oxidant and protease burden in the airways after smoking cessation. *BMC Pulmonary Medicine*.

[44] Yang SC (1993). Relationship between smoking habits and lung function changes with conventional spirometry. *Journal of the Formosan Medical Association*.

